# Deep cerebral venous thrombosis mimicking influenza-associated acute necrotizing encephalopathy: a case report

**DOI:** 10.1186/s13256-017-1444-7

**Published:** 2017-09-26

**Authors:** Daisuke Taniguchi, Sho Nakajima, Arisa Hayashida, Takuma Kuroki, Hiroto Eguchi, Yutaka Machida, Nobutaka Hattori, Hideto Miwa

**Affiliations:** 1 Department of Neurology, Juntendo University Nerima Hospital, 3-1-10 Takanodai, 177-8521 Tokyo, Nerima Japan; 2 Department of Neurology, Juntendo University School of Medicine, 1-21-1 Hongo, 113-0033 Bunkyo, Tokyo Japan

**Keywords:** Influenza, Acute necrotizing encephalopathy, Deep cerebral venous thrombosis, Thalamus, Case report

## Abstract

**Background:**

Acute necrotizing encephalopathy is one of the most devastating neurological complications of influenza virus infection. Acute necrotizing encephalopathy preferentially affects the thalamus bilaterally, as does deep cerebral venous thrombosis, which can lead to misdiagnosis.

**Case presentation:**

A 52-year-old Japanese woman infected with seasonal influenza B virus presented to the emergency care unit in our hospital with progressive alteration of her level of consciousness. Bilateral thalamic lesions were demonstrated by magnetic resonance imaging, leading to a tentative diagnosis of acute necrotizing encephalopathy. However, she had deep cerebral venous thrombosis, and the presence of diminished signal and enlargement of deep cerebral veins on T2*-weighted imaging contributed to a revised diagnosis of deep cerebral venous thrombosis. Anticoagulant therapy was initiated, leading to her gradual recovery, with recanalization of the deep venous system and straight sinus.

**Conclusions:**

To the best of our knowledge, these results represent the first report of deep cerebral venous thrombosis associated with influenza infection. It is clinically important to recognize that deep cerebral venous thrombosis, although rare, might be one of the neurological complications of influenza infection. In the presence of bilateral thalamic lesions in patients with influenza infection, deep cerebral venous thrombosis should be considered in addition to acute necrotizing encephalopathy. Delays in diagnosis and commencement of anticoagulant therapy can lead to unfavorable outcomes.

## Background

Neurological complications of influenza-virus infection are not frequent. When they do occur, they frequently result in severe neurological sequelae with high mortality [1]. One of the most devastating neurological complications of influenza-virus infection is acute necrotizing encephalopathy (ANE) [2]. To date, publications relating to ANE have been limited to case reports and small case series, and the exact prevalence and incidence of ANE remain undetermined [2–8]. ANE manifests with fever, alterations of consciousness, and seizures a few days after the onset of respiratory symptoms [2]. Neuroimaging studies are typically performed, and the results, such as multifocal, symmetrical brain lesions preferentially affecting the thalamus bilaterally, can help to make a prompt diagnosis of ANE [3]. Prompt diagnosis and early commencement of treatment are important to obtain positive outcomes in patients with ANE. However, the results of neuroimaging studies should be cautiously interpreted, to avoid misdiagnosis. For example, the neuroimaging features of deep cerebral venous thrombosis (DCVT) may sometimes be shared by ANE, because thrombosis of the internal cerebral veins, the basal veins, and the great cerebral vein eventually lead to venous (hemorrhagic) infarction and vasogenic edema of bilateral thalami [9]. Diagnosis of DCVT is often delayed because its clinical manifestations (headache, altered consciousness, mental troubles, and motor deficits) are nonspecific and variable [10].

We now report a case in which an initial misdiagnosis of ANE associated with influenza-virus infection was corrected to a diagnosis of DCVT. Although ANE is a life-threatening condition that is associated with influenza-virus infection, the alternative diagnosis of DCVT should be considered because neuroimaging findings for both conditions can be similar.

## Case presentation

A 52-year-old Japanese woman presented to the emergency unit of our hospital with headache and progressive alteration of her level of consciousness. Her medical and family history was unremarkable. Five days before admission, she had developed a high fever and cough, and was diagnosed with infection by influenza B virus following testing of a nasal swab. She received treatment with a single oral inhalation of 40 mg laninamivir octanoate hydrate, a neuraminidase inhibitor.

**Fig. 1:**
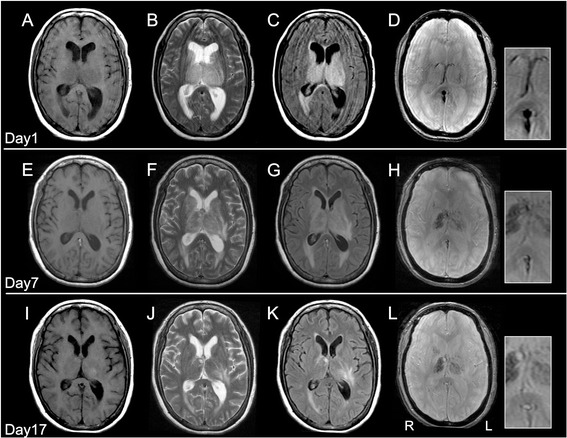
Brain magnetic resonance images. Magnetic resonance imaging was performed at admission (**a**–**d**), and 7 days (**e**–**h**), and 17 days (**i**–**l**) after admission. At admission, the thalamus and caudate nuclei were involved bilaterally with edematous swelling, demonstrated on magnetic resonance imaging by **a** hypointensity of T1-weighted signal, **b** hyperintensity of T2-weighted signal, and **c** hyperintensity of fluid-attenuated inversion recovery signal. **d** Diminished signal and enlargement of the deep cerebral veins and the straight sinus are demonstrated in a T2*-weighted image. **e**–**g** At day 7 after admission, small hyperintense areas were observed in the anterior and left posterior regions of the thalamus in a T1-weighted image (**e**), and a worsening of brain swelling was demonstrated in T2-weighted (**f**) and fluid-attenuated inversion recovery signal (**g**) images. **h** A T2*-weighted image showed hemorrhagic changes in the thalamus with right-side predominance, but abnormal signal of deep cerebral veins was less prominent than at admission. **i**–**k** At day 17, although the size of the T1-hyperintensity area in the left thalamus had increased from day 7 (**i**), the edematous swelling improved (**j**, **k**). **l** Abnormal signal of deep cerebral veins was not present in a T2*-weighted image

**Fig. 2:**
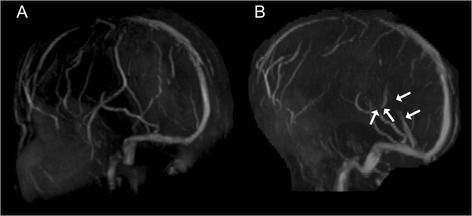
Brain magnetic resonance venography. **a** Brain magnetic resonance venography 7 days after admission showed an occlusion of the internal cerebral vein, the great vein of Galen, and the straight sinus. **b** Magnetic resonance venography 17 days after admission demonstrated recanalization of the deep cerebral veins (*white arrows*)

**Fig. 3:**
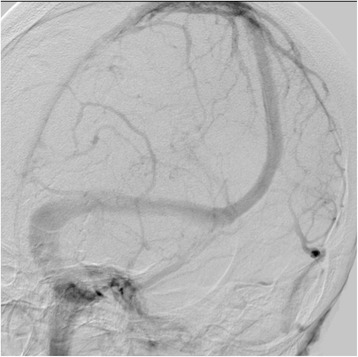
Venous phase of cerebral angiography 7 days after admission. An almost complete lack of filling of the internal cerebral vein, the great vein of Galen, and the straight sinus was demonstrated

On admission, a neurological examination revealed that she was stuporous, with a Glasgow Coma Scale score of E2V4M6. Her cranial nerves were intact. She had no weakness, ataxia, sensory disturbance, or signs of meningeal irritation. Blood tests revealed the presence of iron deficiency anemia (hemoglobin 7.8 g/dl, mean corpuscular volume 63.2 fl, iron 11 mg/dl), slightly elevated platelet counts (434 × 10^3^/μl), and an elevated level of D-dimer (3.9 μg/ml). Her white blood cell counts were within normal ranges. Her level of C-reactive protein was 0.97 mg/dl. Her serum interleukin-6 (IL-6) was elevated at 43.9 pg/ml compared with the reference range of < 4.5 pg/ml. Results indicated that her renal function, liver function, and levels of serum electrolytes were normal. A cerebrospinal fluid (CSF) examination revealed a high opening pressure (300 mmH_2_O), xanthochromia with high red cell count (1820 cells per μl), elevated protein levels (622.2 mg/dl), and normal white cell count (4 cells per μl). CSF bacterial, fungal, and mycobacterial cultures were performed, with negative results. Polymerase chain reaction assays of CSF for herpes simplex virus, varicella zoster virus, Epstein–Barr virus, and cytomegalovirus all had negative results. Brain magnetic resonance imaging (MRI) showed bilateral thalamic lesions, with involvement of bilateral caudate nuclei and the left internal capsule (Figure 1a–c). T2*-weighted MRI revealed diminished signal with an enlargement of deep cerebral veins (Figure 1d). On the basis of the presence of bilateral thalamic lesions, a tentative diagnosis of ANE associated with influenza B infection was made, and treatment with intravenous administration of methylprednisolone (1000 mg per day for 3 consecutive days), immunoglobulins (1 g/kg per day for 1 day), and peramivir (600 mg per day for 1 day) was immediately initiated. Heparin (10,000 units per day) was also administered intravenously to prevent thrombotic events. Despite these treatments, her level of consciousness progressively deteriorated to give a Glasgow Coma Scale score of E1V1M4. Intubation was required 5 days after admission. A brain MRI scan, performed 7 days after admission, demonstrated a worsening of edema (Figure 1e–g). T2*-weighted MRI showed thalamic hemorrhage, and the enlargement of deep cerebral veins was less prominent than at the initial MRI (Figure 1h). Magnetic resonance venography (MRV) 7 days after admission indicated the presence of DCVT (Figure 2a), which was confirmed by cerebral angiography, demonstrating an almost complete lack of filling of the internal cerebral vein, the great vein of Galen, and the straight sinus (Figure 3). Because drainage through the basal vein of Rosenthal and the superficial cerebral veins was observed, we did not attempt invasive therapeutic procedures, and continued treatment with intravenously administered heparin, followed by warfarin. Treatment with intravenously administered glycerol (400 mg per day) was also continued because we presumed that disturbed level of consciousness was partly due to elevated intracranial pressure, although intracranial pressure monitoring was not performed. Elevated doses of heparin (38,000 units per day) and warfarin (5 mg per day) were required for therapeutic efficacy, and our patient developed pulmonary embolism and deep venous thrombosis despite the antithrombotic treatment. However, levels of factors that potentially cause a hypercoagulable state, such as protein C, protein S, antithrombin III, and antiphospholipid antibodies were within normal ranges. In addition, screening with whole-body computed tomography was negative for the presence of cancer.

**Fig. 4:**
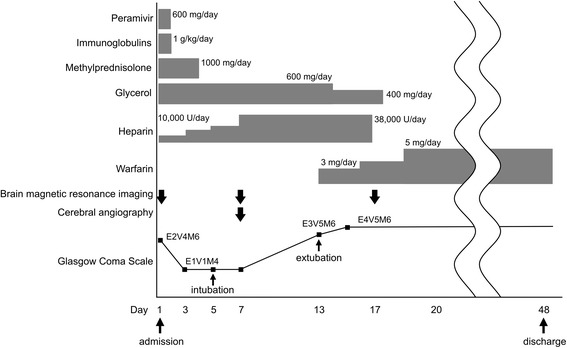
Time course of clinical symptoms, treatment and examination

With the optimization of anticoagulant therapy, the level of consciousness of our patient gradually improved. Follow-up brain MRI, performed 17 days after admission, showed a regression of swelling (Figure 1i–k), disappearance of abnormalities of the deep cerebral veins on T2*-weighted images (Figure 1l), and recanalization of the deep venous system and straight sinus (Figure 2b). She continued to recover, and 6 weeks after admission she was discharged, with the residual symptoms of slight memory impairment and mild right-sided hemiparesis. The time course of clinical symptoms, treatment, and MRI findings is shown in Figure 4.

## Discussion

The patient described in this study developed DCVT during the course of infection with influenza virus B, although initially the neurological complication was identified and treated as ANE. In general, thrombotic vascular events are not frequent occurrences among the wide variety of complications of seasonal influenza infections [11]. However, reports from case studies and cohort studies have described venous and arterial thrombotic events in patients infected with the pandemic influenza A (H1N1) in 2009, including venous thrombosis of upper and lower extremities, arterial thrombosis of the abdominal aorta, pulmonary embolism, myocardial infarction, and cerebral infarction [12, 13]. Cerebral venous thrombosis is an extremely rare complication, and to the best of our knowledge only one prior case of transverse sinus thrombosis has been reported [14]. Whether influenza virus infection can promote thrombotic events is controversial [12], although risk factors for thrombosis are known to include infection by bacteria and also viruses, including herpesviruses, hepatitis viruses, parvovirus B19, and human immunodeficiency virus [15]. Pathophysiological mechanisms that specifically drive thrombosis during infection have not yet been fully elucidated, but inflammation triggered by viral infection is regarded as one of the causes of the hypercoagulable state [12, 16]. Notably, in our patient, a high serum level of IL-6 was found, suggesting that inflammation resulted in DCVT. Iron deficiency anemia was also observed in our patient, and is known to contribute to the induction of a hypercoagulable state [17].

DCVT presents with a wide range of symptoms and modes of onset, which can complicate diagnosis of this condition [10]. The identification of bilateral thalamic lesions by MRI can provide an important clue to diagnosis of DCVT [9, 18], but these lesions are also observed in ANE, which is a contributing factor in the misdiagnosis of DCVT [3]. On clinical examination, it is by no means easy to clearly distinguish ANE from DCVT, particularly if the patient has an influenza infection, because both ANE and DCVT present with nonspecific symptoms such as alteration of consciousness. Epidemiologically, ANE generally involves pediatric patients and is associated with influenza A virus. However, ANE can also occur in adult patients and can be caused by influenza B virus [4, 6]. With regard to laboratory findings, an elevation of the level of D-dimer, which is found in most patients with DCVT, might be expected to be a strong clue to diagnosis of DCVT; however, this result is observable nonspecifically in a wide variety of frequently encountered conditions, such as dehydration or prolonged bed rest [10]. Instead, CSF findings may be helpful to differentiate DCVT from ANE. Although both DCVT and ANE are associated with normal white cell counts in CSF, xanthochromia with a high red-cell count, presumably resulting from disruption of the blood–brain barrier due to venous infarction, has been reported in previous case reports of cerebral venous thrombosis [19, 20], but not in previous cases of ANE [2]. On pathophysiologic examination, vasogenic edema, hemorrhage, and necrosis in the thalamic lesions are common features of both DCVT and ANE, which result in similar neuroimaging findings [3]. Thus, careful and detailed MRI studies are necessary to make prompt differential diagnoses. In our patient, observation of diminished signal and enlargement of deep cerebral veins on T2*-weighted MRI led us to reconsider the diagnosis of ANE. The current standard methods for making a diagnosis of DCVT are cerebral angiography, MRV, and computed tomography angiography. Meanwhile, recent studies have highlighted the clinical benefit of T2*-weighted imaging of DCVT, in which the thrombosis gives a hypointense signal [21, 22]. This finding is detectable in ~ 90% of sites of DCVT at the first MRI investigation, suggesting that T2*-weighted imaging is valuable for clot detection in DCVT in conjunction with conventional MRI, especially in the acute phase of thrombosis [22].

Guidelines for treatment of DCVT recommend dose-adjusted intravenous administration of heparin, followed by oral anticoagulants [10]. In addition, if intracranial pressure is severely raised, treatment with osmotic agents is recommended [10]. By contrast, treatments for ANE, including intravenous administration of steroids and immunoglobulins, potentially cause hypercoagulability, which might lead to exacerbation of DCVT. Therapeutically, the differential diagnosis of ANE and DCVT is very important. In our case study, after initial misdiagnosis of ANE, T2*-weighted imaging helped in the diagnosis of DCVT, which led to appropriate treatment, recanalization, and a positive outcome for our patient.

## Conclusions

It is clinically important to recognize that DCVT, although rare, might be a neurological complication of influenza virus infection. If bilateral thalamic lesions are identified in patients with influenza infections, DCVT as well as ANE should be kept in mind, and T2*-weighted imaging could be a useful sequence for discriminating DCVT from ANE.

## Abbreviations

ANE: Acute necrotizing encephalopathy
CSF: Cerebrospinal fluid
DCVT: Deep cerebral venous thrombosis
IL-6: Interleukin-6
MRI: Magnetic resonance imaging
MRV: Magnetic resonance venography
